# Legumain Promotes Gastric Cancer Progression Through Tumor-associated Macrophages *In vitro* and *In vivo*

**DOI:** 10.7150/ijbs.36467

**Published:** 2020-01-01

**Authors:** Hongbin Wang, Binghong Chen, Yingying Lin, Yi Zhou, Xiaobo Li

**Affiliations:** 1 Division of Gastroenterology and Hepatology, Key Laboratory of Gastroenterology and Hepatology, Ministry of Health, Shanghai Institute of Digestive Disease, Renji Hospital, School of Medicine, Shanghai Jiao Tong University, Shanghai, China.; 2 Department of Gastroenterology, Punan Hospital, Pudong New Area, Shanghai, China.; 3 Department of Neurosurgery, Renji Hospital, School of Medicine, Shanghai Jiao Tong University, Shanghai, China.

**Keywords:** Legumain, gastric cancer, tumor-associated macrophages, angiogenesis

## Abstract

Tumor-associated macrophages (TAMs) play a crucial role in the tumor microenvironment. Legumain (LGMN) has been shown to be a tumor-promoting protein, but the effect of LGMN on TAMs in the progression of gastric cancer (GC) is under exploration. Our studies included the construction of LGMN-knockdown and LGMN-overexpressing TAMs induced from the human cell line THP-1 (PMA/IL-4/IL-13) and murine cell line Raw264.7 (IL-4/IL-13). A CCK-8 assay and transwell migration assay indicated that upregulation of LGMN expression in TAMs stimulated cell proliferation, migration and invasion *in vitro,* while downregulation of LGMN expression reduced cell proliferation, migration and invasion. *In vivo* experiments revealed slower growth, less angiogenesis, and less Ki67 expression in LGMN-knockdown TAMs injected with gastric cancer cells compared to control TAMs injected with GC cells. Together, these study results suggested that LGMN^+^ TAMs*,* which may serve as a potential target for GC treatment, promoted gastric cancer cell proliferation and angiogenesis *in vitro* and* in vivo*.

## Introduction

There are pronounced global epidemiological variations in gastric cancer (GC) incidence, with higher incidence rates in East Asia, Eastern Europe and South America than in the rest of the world 1. Globally, gastric cancer ranked fifth in cancer incidence and second in mortality, and there were 984 000 incident cases of GC and 841 000 deaths in 2013 2. Population growth and aging resulted in a larger number of cases of stomach cancers in 2013 compared with 1990; although part of this increase was offset by falling rates, GC is still the major cancer worldwide, comprising the global disease burden 2-4. In areas where GC screening methods (such as endoscopic screening) are not universal, GC is often found at an advanced stage by nonspecific symptoms 5, 6, which leads to poor overall survival. Tumor metastasis is a key factor affecting the prognosis of GC; for metastatic patients, systemic chemotherapy with or without biological agents represents the standard of care, contributing to improved overall survival and an improved quality of life 7, 8.

Macrophages are one of the most abundant inflammatory cells in the tumor microenvironment 9, 10. Substantial evidence suggests that macrophages adopt a protumorigenic phenotype rather than a tumoricidal phenotype and function as a vital driver of tumor-promoting inflammation, leading to tumor progression by various methods including promoting genetic instability, facilitating metastasis, supporting cancer stem cells and inhibiting protective immunity 11-13. Legumain (LGMN), also known as asparagine endopeptidase (AEP), is a lysosomal cysteine protease originally identified in the seeds of legumes, which is also present in the human body and is associated with a variety of tumor types at the stages of development, invasion and metastasis 14-16. Numerous studies have confirmed that LGMN is a potential prognostic factor in GC 17, 18. What is the role of LGMN in macrophages in the progression and metastasis of GC? The present study explored the relationship of LGMN expressed by macrophages with GC metastasis *in vitro* and *in vivo*.

## Materials and Methods

*Cell culture.* Human acute monocytic leukemia cells, THP-1 cells (Cat. CBP60518, Cobioer, Nanjing, China), and mouse macrophages, RAW 264.7 cells (Cat. CBP60533, Cobioer, Nanjing, China), were cultured in Dulbecco's modified Eagle's medium (HyClone; GE Healthcare) supplemented with 10% fetal bovine serum (Gibco; Thermo Fisher Scientific, Inc.) and maintained in a humidified atmosphere at 37°C with 5% CO_2_. THP-1 cells were activated and differentiated into macrophages by incubation with phorbol-12-myristate-13-acetate (PMA; 100 ng/ml in complete medium) and IL-4/IL-13 for 3 days, while RAW 264.7 cells were treated with IL-4/IL-13 only. The culture medium was exchanged every day.

*Lentiviral vector-mediated gene overexpression or knockdown.* An LGMN overexpression sequence was constructed by Hanyin Ltd., Co (Shanghai, China). A recombinant lentivirus and negative control (NC) lentivirus were prepared and titered to 10^9^ transfection units/ml. After 48 h, the efficiency of overexpression was confirmed via RT-qPCR. To obtain stably transfected cells (LGMN-OE), macrophages were seeded in six-well dishes at a density of 1 x 10^5^ cells per well. The cells were then infected with the same virus titer on the following day and treated with 8 μg/ml polybrene. At 72 h post-viral infection, the culture medium was replaced with a selection medium containing 4 μg/ml puromycin. The puromycin-resistant cells were amplified in a medium containing 2 μg/ml puromycin for 7 days and then transferred to a medium without puromycin. To downregulate the expression of LGMN in both macrophage cell lines, two different LGMN shRNA sequences were cloned into the pTRIPZ plasmid (Open Biosystems, RHS4750, Huntsville, Alabama, USA) according to the manufacturer's instructions. An shRNA sequence targeting LGMN was cloned into the plvx-shRNA plasmid. A non-silencing lentiviral shRNA vector was used as a control. The lentiviruses were packaged using psPAX2 and pMD2G, a three-plasmid system. To obtain stable cell lines, lentivirus supernatant was added to THP-1 and Raw264.7 cells, followed by screening with 1 μg/ml puromycin for 2 weeks. The expression of LGMN was downregulated in these cell lines when the cells were treated for longer than 4 days with 1 *μ*g/ml doxycycline (Dox), an analog of tetracycline, in the culture medium. To overexpress LGMN in THP-1 and Raw264.7 cells, LGMN was cloned into the pLVX-IRES-ZsGeen1 plasmid. Lentivirus supernatant was added to the culture medium of THP-1 and Raw264.7 cells. The infection rate was assessed using a fluorescence microscope.

*Western blot analysis.* Total protein was extracted from cells with a cell lysis buffer (50 mM Tris-HCl pH 8.0, 120 mM NaCl, 0.5% NP-40, and 1 mM PMSF) and evaluated by BCA methods. Protein (30 μg) was subjected to 10% SDS-polyacrylamide gel electrophoresis and then transferred to polyvinylidene difluoride membranes (EMD Millipore, Billerica, MA, USA). The membranes were incubated with a blocking buffer (5% skim milk in TBS-T) at room temperature for an hour. After that, the membranes were incubated with the following antibodies at a 1:500 dilution overnight at 4°C: an anti-LGMN antibody (cat. no. 67017-1-Ig; ProteinTech Group, Inc., Chicago, IL, USA) and an anti-β-actin antibody (cat. no. 4970; Cell Signaling Technology, Inc., Beverly, MA, USA). The membranes were washed with TBS-T and then incubated with a horseradish peroxidase-conjugated anti-rabbit or anti-mouse antibody (1:10,000 dilution; Sigma-Aldrich; Merck KGaA, Darmstadt, Germany) at room temperature for 2 h. Detection was performed using western blot detection reagents (Odyssey; LI-COR Biosciences, Lincoln, NE, USA).

*Cell proliferation and motility or invasion assays* A Cell Counting Kit-8 (CCK-8) assay was performed to assess cell proliferation. Briefly, transfected PMA-treated THP-1 and RAW 264.7 cells were plated at a density of 1 × 10^4^ cells/well in a 96-well plate. Then, 10 μL of CCK-8 solution was added to each well and incubated for 2 h. Next, absorbance values were detected at a wavelength of 450 nm using a Bio-Rad microplate reader. Cell viability was expressed as the optical density (OD) values of the treated groups/OD values of the control groups × 100%.

A Transwell migration assay was employed to evaluate cell invasion. 24-well Transwell plates with 8-μm-diameter filters (Coring, NY, USA) were utilized. Approximately 2×10^5^ cells suspended in 200 μl of serum-free medium were placed in the upper chamber, and 750 μl of 10% FBS medium was added to the lower chamber. The plate was incubated for 8 h at 37 °C with 5% CO_2_. Then, the cells on the upper side were carefully removed with a cotton swab. The cells that passed through the filter were fixed in 40 g/L methanol for 15 min and then stained with 0.1% crystal violet for 15 min. The cells on the filters were examined and counted under an inverted microscope. Each experiment was repeated three times.

*Xenograft tumor model in nude* mice. A total of 5 x 10^5^ human gastric cancer SGC7901 cells together with 1 x 10^5^ TAMs induced from THP-1 cells (PMA/IL-4/IL-13) with or without LGMN KD were subcutaneously transplanted into the right flank of nude mice (n=6 in each group, 4 to 6 weeks old, male, purchased from the Department of Experimental Animal Science, Shanghai Jiao Tong University School of Medicine) using a 1-ml syringe. Tumor size was measured using a Vernier caliper every three days. The animals were sacrificed, and the masses were processed, followed by hematoxylin-eosin (H&E) staining. The animal study was approved by the Institutional Animal Care and Use Committee of Renji Hospital Affiliated to Shanghai Jiao Tong University School of Medicine.

*Immunofluorescence and immunohistochemical assays.* Immunofluorescence experiments were performed. Briefly, 2×10^5^ cells were seeded on coverslips in each well of a 6-well plate. The cells were washed 3 times with PBS before fixation with 4% paraformaldehyde. The cells were blocked with PBS containing 1% goat serum for 30 min. Antibodies were incubated at 4 °C overnight. The cells were washed 6 times with PBS for a total of 3 hours and incubated with secondary antibodies for 1 hour at room temperature. The samples were observed with a Zeiss laser scanning confocal microscope (LSM Meta 510). Single sections are shown. Images were processed (colored and merged) with Zeiss (LSM 510) software. Immunohistochemical assays were performed. An anti-Ki67 antibody (ab8191, Abcam) was used as the primary antibody.

*Statistical analysis.* Values are expressed as the mean ± standard error of the mean. One-way analysis of variance with Tukey's test was performed for comparisons of multiple groups. All statistical analyses were performed using SPSS for Windows v. 17.0 (SPSS, Inc.). A two-tailed *p*<0.05 was considered to indicate a statistically significant difference.

## Results

**Figure 1:**
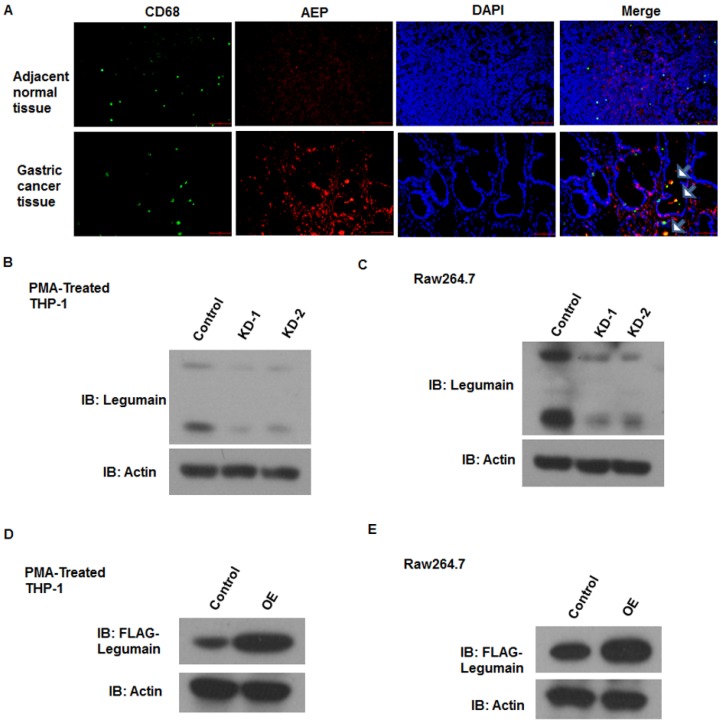
** Efficient knockdown or overexpression of LGMN in monocytes/macrophages. (A)** Expression of LGMN and CD68 in gastric cancer tissue samples and adjacent normal tissue samples. **(B** and** C)** Protein bands for LGMN and β-actin in THP-1 (PMA/IL-4/IL-13) cells and Raw264.7 (IL-4/IL-13) cells with or without LGMN knockdown. **(D** and** E)** Protein bands for FLAG-LGMN and β-actin in THP-1 (PMA/IL-4/IL-13) cells and Raw264.7 (IL-4/IL-13) cells with or without LGMN overexpression.

*Efficient knockdown or overexpression of LGMN in TAMs induced from monocytes/macrophages.* The expression of legumain in TAMs derived from normal or gastric cancer tissue samples was analyzed. As shown in the revised Figure 1a, legumain was highly expressed in TAMs from the gastric cancer tissue samples compared to those from the adjacent normal tissue samples (**Figure 1A**). To obtain monocytes/macrophages with stable knockdown or overexpression of LGMN, lentivirus-mediated gene transfection was applied *in vitro*. A western blot assay was used to detect the efficiency of transfection. The expression of LGMN was significantly knocked down in TAMs induced from THP-1 cells (PMA/IL-4/IL-13) (**Figure 1B**) and Raw264.7 cells (**Figure 1C**). LGMN was significantly overexpressed in THP-1 cells (**Figure 1D**) and Raw264.7 cells (**Figure 1E**).

**Figure 2:**
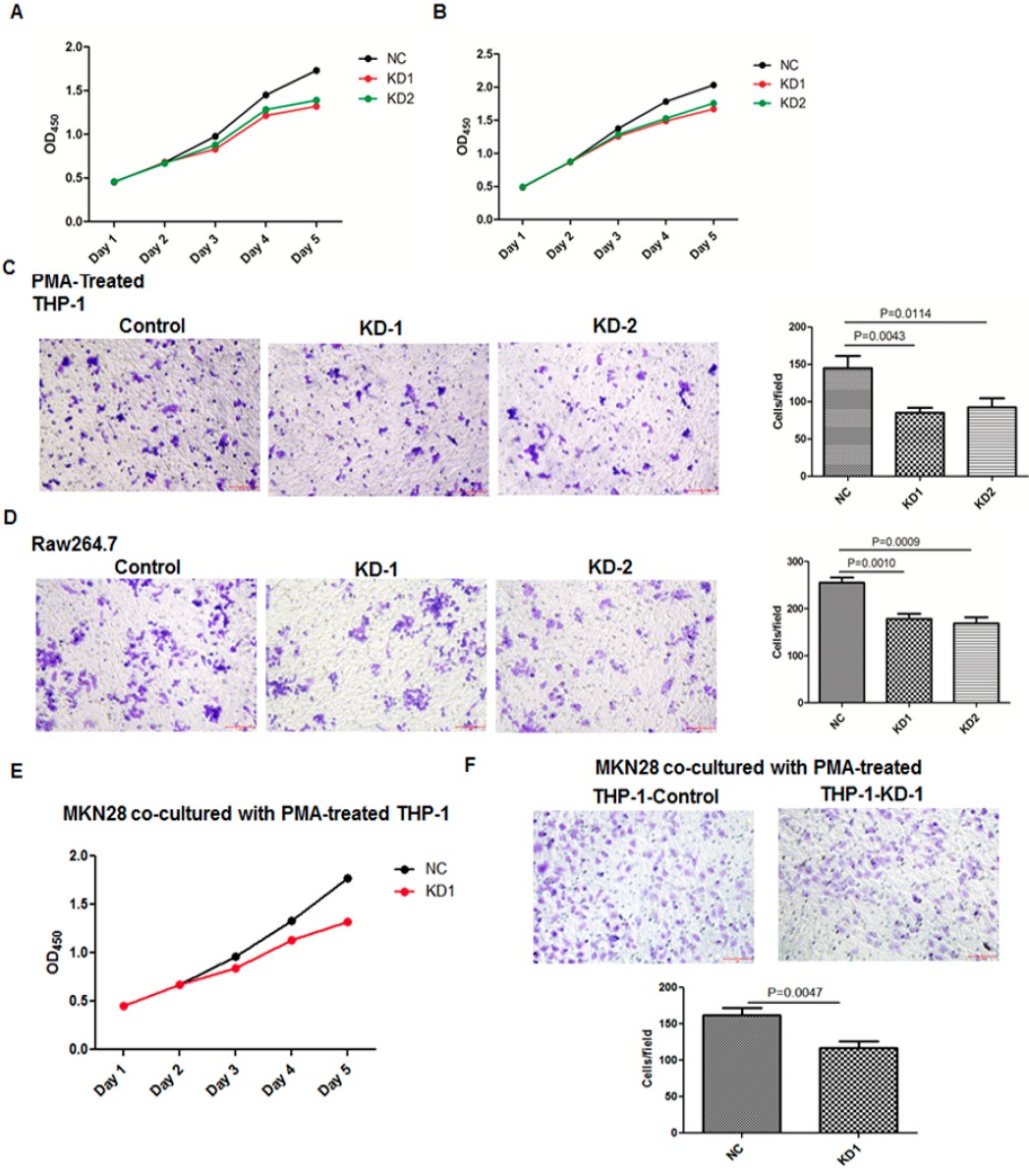
** Knocking down LGMN expression in TAMs reduced their activity and migration. (A** and** B)** Growth curves for THP-1 and Raw264.7 cells with or without LGMN knockdown. **(C** and** D)** Crystal violet staining after a transwell migration assay and statistical analysis of the cells/field for THP-1 and Raw264.7 cells with or without LGMN knockdown. **(E)** Growth curves for MKN28 cells cocultured with THP-1 cells with or without LGMN knockdown. **(F)** Transwell migration assay of MKN28 cells cocultured with THP-1 cells with or without LGMN knockdown.

**Figure 3:**
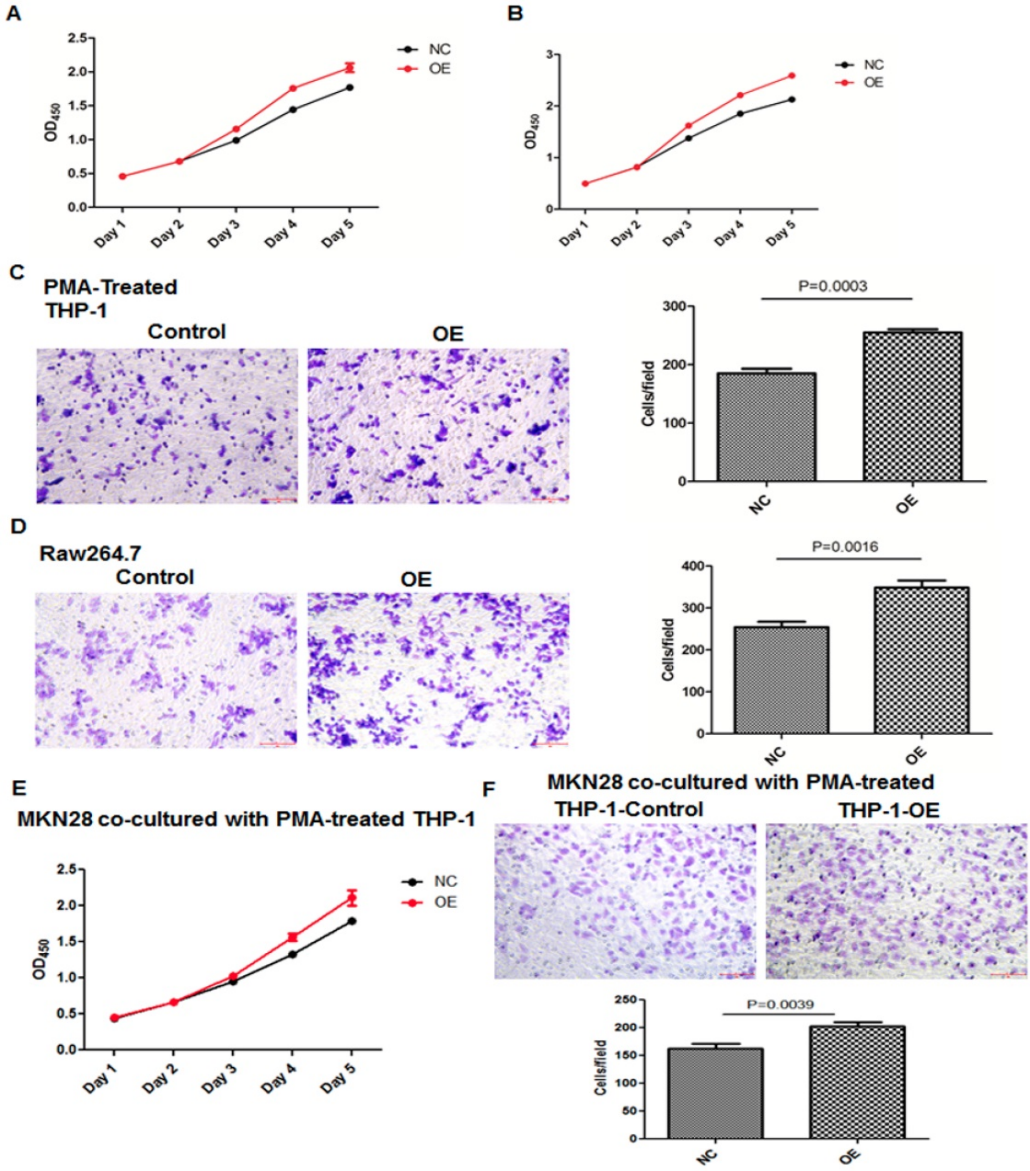
** Overexpression of LGMN in TAMs enhanced their activity and migration. (A** and **B)** Growth curves for THP-1 and Raw264.7 cells with or without LGMN knockdown. **(C** and** D)** Crystal violet staining after a transwell assay and statistical analysis of the cells/field for THP-1 and Raw264.7 cells with or without LGMN overexpression. **(E)** Growth curves for MKN28 cells cocultured with THP-1 cells with or without LGMN overexpression. **(F)** Transwell migration assay of MKN28 cells cocultured with THP-1 cells with or without LGMN overexpression.

*Knockdown or overexpression of LGMN in TAMs affected growth and migration.* To investigate the function of LGMN in GC, we constructed LGMN-overexpressing (OE) or LGMN-knockdown (KD) THP-1 cell lines and Raw264.7 cell lines. According to growth curve results, we found that LGMN depletion* in macrophages* significantly reduced the growth of both cell lines compared with that of negative controls (NCs) (**Figure 2A and B**). In contrast, overexpression of LGMN in *macrophages* dramatically enhanced cell growth when compared with endogenous expression in control cells (**Figure 3A and B**). The results of a transwell migration assay indicated that after LGMN knockdown, the amount of invaded cells was decreased to approximately half that of NC cells in PMA-treated THP-1 cells (**Figure 2C**) and approximately one third that of NC cells in Raw264.7 cells (**Figure 2D**). Overexpression of LGMN significantly increased the number of migrating cells in both cell lines (**Figure 3C and D**).

CCK-8 and transwell migration assays studying GC cancer cells cocultured with TAMs with or without legumain expression were performed. The results showed that the GC cancer cells cocultured with the legumain-knockdown TAMs showed significantly reduced cellular proliferation and migration (**Figure 2E and 2F**). However, the MKN28 GC cancer cells cocultured with the legumain-overexpressing TAMs exhibited significantly increased cellular proliferation and migration (**Figure 3E and 3F**).

**Figure 4:**
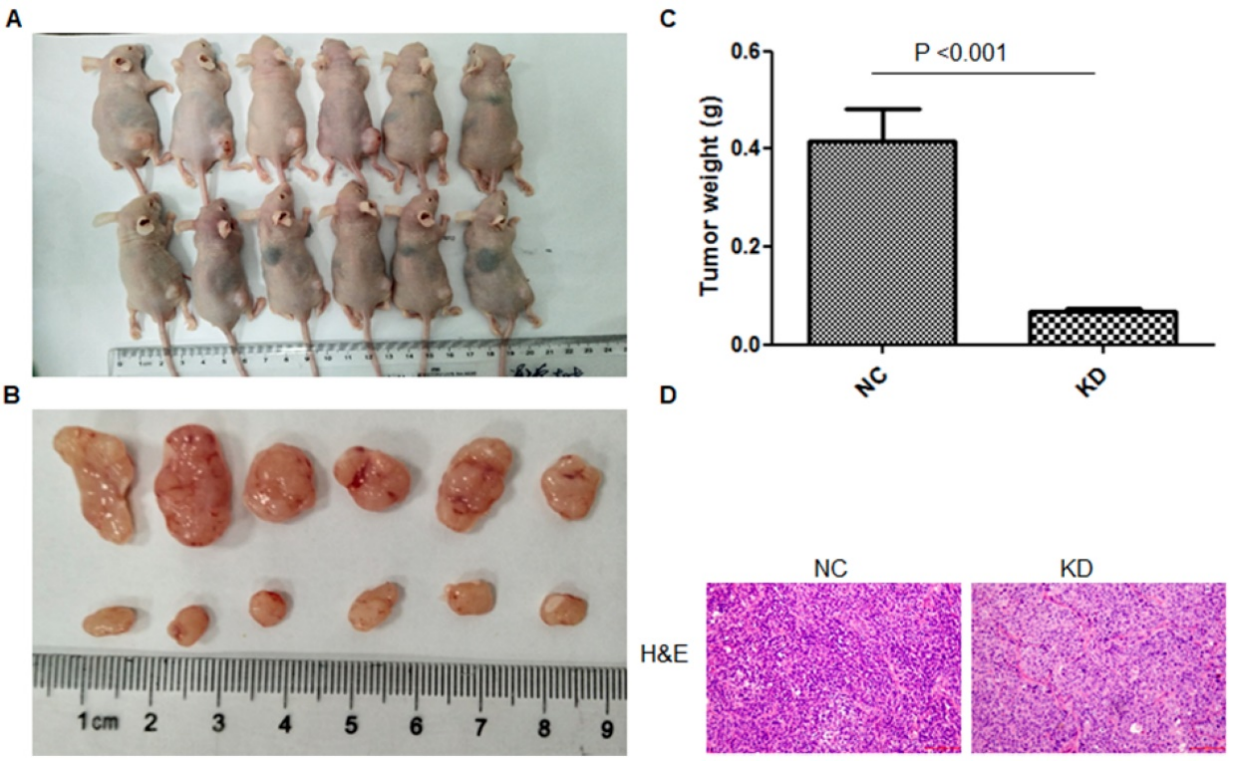
** LGMN-KD TAMs reduced tumor development *in vivo*. (A)** Tumor formation after subcutaneous injection of SGC7901 gastric cancer cells mixed with TAMs induced from THP-1 cells (PMA/IL-4/IL-13) with or without LGMN KD. **(B)** Dissected tumors. **(C)** Tumor weights for the NC and KD groups. **(D)** H&E staining for the NC and KD groups.

*Legumain-suppressed TAMs inhibited tumor progression in vivo.* For further study of the function of LGMN in TAMs* in vivo,* LGMN-suppressed Raw264.7 cells were mixed with GC cells and transplanted into nude mice, and the model was constructed successfully. As shown in **Figure 4A-C,** the size and weight of the tumors in the LGMN-KD group were significantly smaller than those of the tumors in the NC group. H&E staining revealed many more tumor cells and darker stained nuclei in the NC group than in the LGMN-KD group (**Figure 4D**).

**Figure 5:**
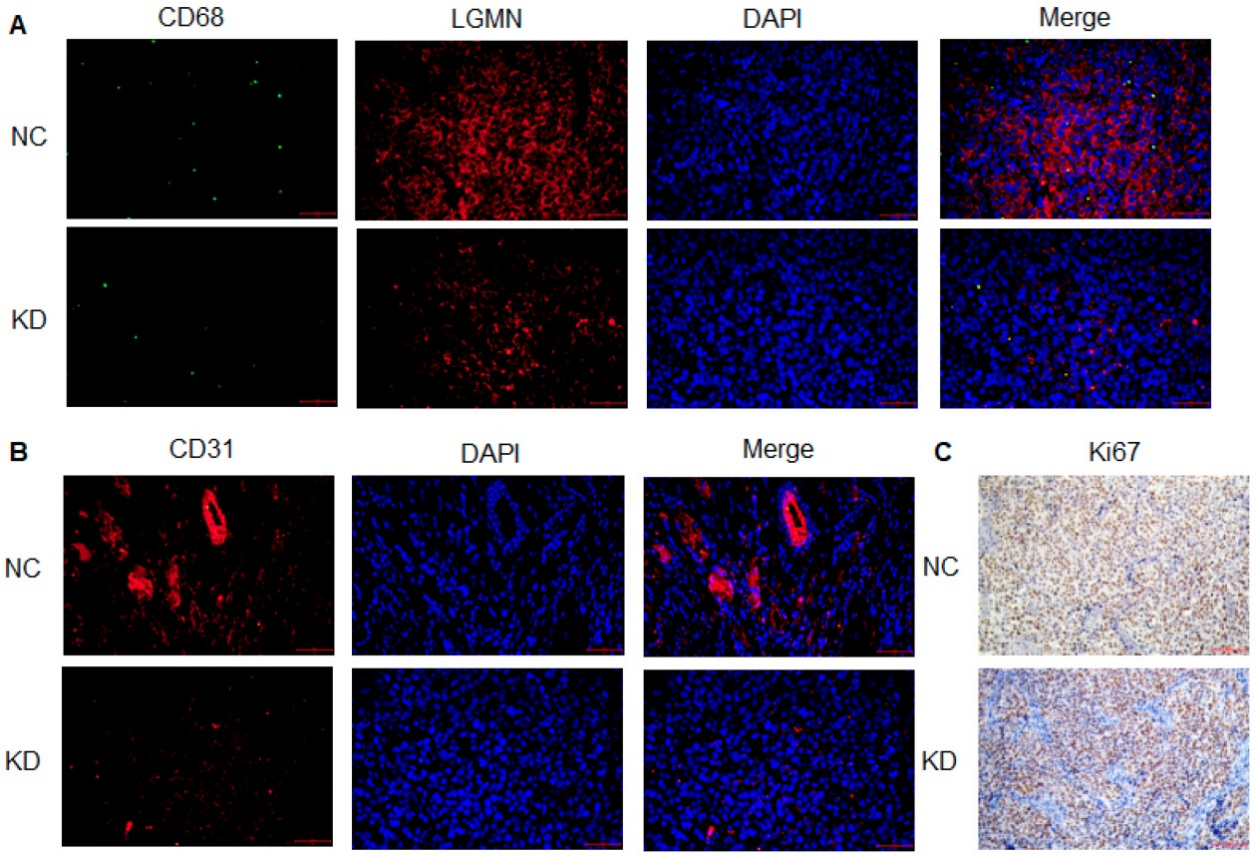
** LGMN-KD TAMs reduced tumor cell proliferation and angiogenesis *in vivo.* (A)** Green fluorescence-labeled CD68, red fluorescence-labeled AEP and DAPI-labeled nuclei in LGMN-NC and LGMN-KD tissue samples. **(B)** Red fluorescence-labeled AEP, DAPI-labeled nuclei and merged images of LGMN-NC and LGMN-KD tissue samples. **(C)** Immunohistochemical staining for Ki67 in LGMN-NC and LGMN-KD tissue samples.

*Legumain-suppressed TAMs reduced tumor growth and angiogenesis in vivo.* Results for immunofluorescence staining showed lower expression of APE in LGMN-suppressed TAM-associated tumors than in NC tumors (**Figure 5A**). Expression of the angiogenesis marker CD31 was clearly suppressed in the LGMN-suppressed TAM-associated tumors (**Figure 5B**). Immunohistochemistry was used to detect Ki67, a biomarker of malignant growth, and the results indicated downregulation of Ki67 expression in the LGMN-suppressed TAM-associated tumors (**Figure 5C**).

**Figure 6:**
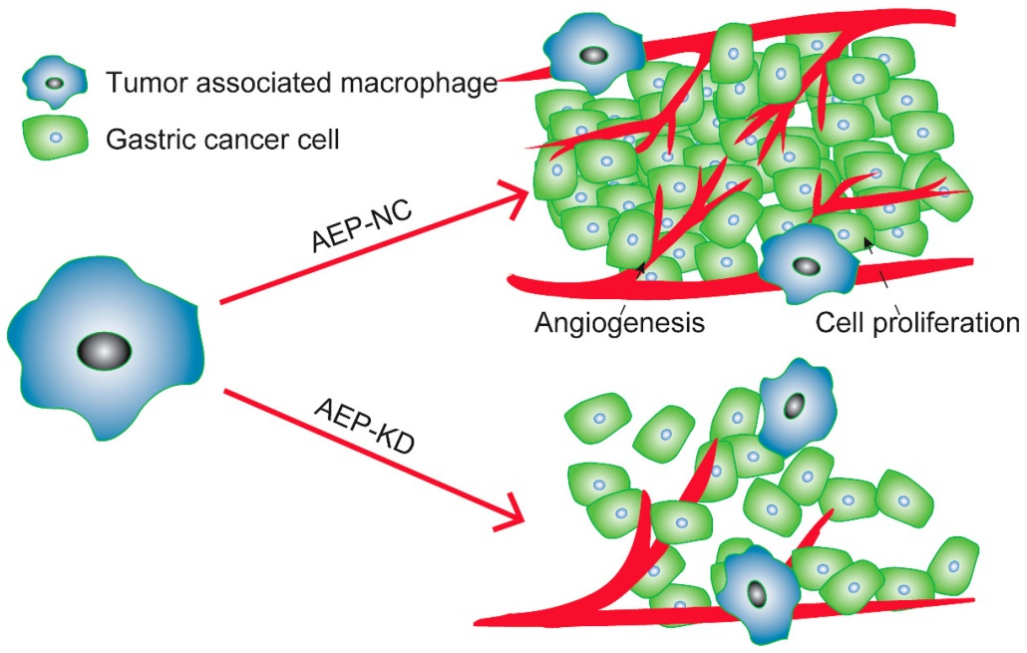
LGMN-KD TAMs reduced tumor cell proliferation and angiogenesis. Diagram of TAMs promoting GC proliferation and angiogenesis.

In summary, LGMN played an important role in TAMs. On the one hand, *in vitro* experiments suggested that altering the expression of LGMN changed the growth and migration of TAMs. On the other hand, *in vivo* experiments proved that LGMN-suppressed TAMs reduced tumor growth and angiogenesis **(Figure 6)**.

## Discussion

The tumor microenvironment is a multifarious niche of cells that evolves with and provides support to tumor cells 19-21. Among the innate and adaptive immune cells recruited to a tumor site, macrophages are particularly abundant and are present at all stages of tumor progression 12, 22. Tumor infiltration by tumor-associated macrophages is one of the predictive factors in gastric cancers correlated with an unfavorable prognosis 23. The mechanisms by which macrophages promote tumors are complex. TAMs may promote angiogenesis and lymphangiogenesis in GC, and these effects may be achieved by enhancing VEGF expression 24. Transfer of TAM-derived miR-21 through exosomes confers cisplatin resistance to gastric cancer cells by activating the PI3K/AKT signaling pathway and downregulating PTEN expression 25. In addition, TAMs promote the epigenetic silencing of gelsolin through DNA methyltransferase 1 activity induced by CCL5/CCR5/STAT3 signaling in gastric cancer cells 26. Previous studies found that CXCL8 was a vital driver of GC and it was secreted by macrophages. In turn, CXCL8 inhibited CD8^+^ T cell functions by inducing the expression of PD-L1 on macrophages, which eventually promoted tumor progression 27. TAMs can exert dual influences on cytoreductive therapies, either antagonizing antitumor activity by orchestrating tumor-promoting activity and the tissue-repair response or enhancing the overall antineoplastic effect directly 28, 29. Currently, immunotherapy shows great potential in cancer treatment; TAMs can express molecular triggers of checkpoint proteins that regulate T cell activation, which are targets of certain checkpoint blockade immunotherapies 30, 31. Other macrophage-associated anticancer therapies are under investigation, such as inhibiting macrophage recruitment and survival in tumors; macrophage re-education to induce an antitumor M1 phenotype; and monoclonal antibodies that elicit macrophage-mediated extracellular killing or phagocytosis and intracellular destruction of cancer cells 11, 12, 32.

LGMN has been proven to be associated with the progression of tumors, and TAMs also play critical roles in promoting angiogenesis, tumor invasion and metastasis. A previous study indicated that tumor-promoting functions may be associated with the expression of LGMN 33-35. Shen L 36 found that the number of M2 TAMs was negatively correlated with the prognosis of diffuse large B cell lymphoma and that overexpression of LGMN in Raw 264.7 cells promoted the formation of stromal vascular endothelium and degradation of fibronectin and collagen I, which together facilitate tumor progression. Moreover, a series of LGMN substrates including MMPs and p53, which are crucial for tumor progression, have been found 37, 38. To further understand the roles of LGMN activity in cancer progression and inflammation, Edgington LE 39 designed an activity-based probe that bound active LGMN to trace changes in LGMN in macrophages; the result indicated that the expression of LGMN was highly correlated with macrophage activation and might be an ideal marker for early metastasis. Our study regulated the expression of LGMN in macrophages, which induced significant changes in the growth and metastasis of GC cells. In addition, *in vivo* experiments suggested that the growth and angiogenesis of gastric cancer tumors were suppressed by knocking down the expression of LGMN in TAMs. Together, these results supported and coincided with the findings of reported studies, indicating that LGMN acts as a very important driving factor in TAMs to promote GC progression.

## Conclusion

In conclusion, the current study suggests that LGMN in TAMs performs a novel oncogenic role in the regulation of gastric tumor cell proliferation and invasion *in vitro* and *in vivo*. Our findings provide new insights into the molecular details of GC-associated macrophages and propose potential therapeutic targets for this disease.

## Abbreviations

TAMs: tumor-associated macrophages
LGMN: legumin
AEP: asparagine endopeptidase
GC: gastric cancer
NC: negative control
OE: overexpression
TBST: Tris-Buffered Saline and Tween 20
CCK-8: A Cell Counting Kit-8 assay
